# Enhancing rock phosphate integration rate for fast bio-transformation of cow-dung waste-paper mixtures to organic fertilizer

**DOI:** 10.1186/s40064-016-3497-2

**Published:** 2016-11-16

**Authors:** F. O. Unuofin, M. Siswana, E. N. Cishe

**Affiliations:** 1 Department of Applied Science, Walter Sisulu University, 19 Manchster Road, Chiselhurst, P.O. Box 19712, East London, South Africa; 2 Development and Innovation, Walter Sisulu University, Nelson Mandela Drive, PBX1, Mthatha, 5099 South Africa

**Keywords:** Humic acid, Germination index, Microbial biomass carbon, Vermicompost, Carbon to nitrogen ratio, Rock phosphate

## Abstract

Rock phosphate (RP) addition in cow-dung waste-paper mixtures at rates above 2% P has been reported to increase the rate of bio-transformation and humification of organic waste mixtures during vermicomposting to produce organic fertilizer for organic farming. However, the optimization of RP for vermicomposting was not established. The objective of this study was to determine the optimal amount of RP integration rates for effective bio-transformation of cow-dung waste-paper mixtures. Arrays of RP integration degrees (0, 0.5, 1, 1.5, 2, and 4% P as RP) were thoroughly mixed with cow- dung waste-paper mixtures to achieve an optimized C:N ratio of 30 and allowed to vermidegrade following the introduction of earthworms at a stocking mass of 12.5 g-worms kg^−1^. The bio-transformation of the waste mixtures was examined by measuring C:N ratios and humification index (HI) and per cent ash and volatile solids. Application of 1% P as RP resulted in fast bio-transformation and maturation of cow-dung waste-paper mixtures. A scanning electron microscopy (SEM) was used to evaluate the morphological properties of the different vermicomposts affected by rates of RP showing the degree of degradation of initial compacted aggregates of cellulose and protein fibres in the mixtures at maturity. A germination test was used to further determine phytotoxicity of the final composts and microbial biomass assessment. The final vermicompost (organic fertilizer) had a C:N ratio of 7, MBC of 900 mg kg^−1^ and HI of 27.1%. The RP incorporation rate of 1% P of RP investigated is therefore, recommended for efficient vermidegradation and humification of cow-dung waste-paper mixtures. However, higher rates of RP incorporation should be considered where greater P enrichment of the final vermicompost (organic fertilizer) is desired.

## Background

Vermicomposting has proved to be a suitable technique of processing biodegradable organic wastes and converting them to organic fertilizers, because of its low cost and the large quantity of wastes that can be processed (Lim et al. 2016; Wu et al. 2014). Generally, the vermicompost which is produced from vermicomposting process can enhance soil fertility physically, chemically and biologically (Lim et al. 2015). However, the nutritional values of some compost/vermicompost products are erratic and determined principally by the types of the substrates used and the degree of composting (Matiullah and Muhammad 2012; Mupondi 2010; Yan et al. 2012). In order to increase the acceptability of vermicompost products as sources of nutrients or as growing media, it is essential to increase their P contents. Biswas and Narayanasamy (2006) found that it is possible to increase the total P content of straw compost from 0.37% in control to 2.20% by adding 4% P as RP, while (Yan et al. 2012) also increased the total P of rice straw vermicompost from 0.392 to 0.82% by adding 2% P as RP. Matiullah and Muhammad (2012) also increased the total P content in poultry litter from 0.3 to 1.02% by adding 4% P as RP.

In addition to increasing the P contents of composts, the inclusion of RP has also been shown to improve the humification of organic wastes. Singh and Amberger (1990) reported that organic solid waste compost inoculated with RP not only increased its P content but also enhanced the humification of the resultant vermicomposts. Similarly, Mishra (1992) obtained highly humified compost from plant wastes composting by adding 25% Mussoorie RP. This prompted (Mupondi 2010) to investigate the effect of RP incorporation on the vermicomposting of cow-dung paper-waste mixtures. He investigated rates of RP incorporation ranging from 2 to 8% P as RP. The results showed that the vermicomposts were highly humified and had high total P and N contents as well as available P and N contents compared to the control. All rates of RP incorporation >2% P improved humification to more or less the same extent suggesting that lower rates of RP incorporation could possibly being effective in improving the vermidegradation of cow dung waste paper mixtures. This study, therefore, investigated the effectiveness of using low RP application rates of less than 2% P or 4% P in improving the vermidegradation of cow-dung paper-waste mixtures.

The objectives of this study were to determine: (1) the minimum amount of rock phosphate necessary for efficient bio-transformation of cow-dung paper-waste mixtures, and (2) the effects of the rate of RP application on total P, Bray-1 extractable P, microbial biomass index, Ammonium and nitrite-nitrate levels and germination index of the vermicompost (organic fertilizers).

## Methods

### Location description, earthworms and wastes utilized

The vermicomposting experiment was performed in an enclosed, shaded yard at the University of Fort Hare’s Teaching and Research farm located 32°46′S and 26°50′E in the Eastern Cape Province of South Africa under an ambient mean temperature of 25 °C. Paper waste used for the study was collected from the University printing press (Xerox) and Faculty offices, while RP was obtained from Phalaborwa in Limpopo Province, South Africa, which is granitic in nature. *Eisenia fetida* (*E. fetida*) earthworms used in the study were collected from a local wormery at the University of Fort Hare’s Teaching and Research farm. Cow dung was obtained from Keiskammahoek Dairy Project located about 60 km North East of the University of Fort Hare.

### Experimental method

#### Phosphorus enhancement of vermicomposts with RP

Rock phosphate was incorporated into cow dung waste paper mixture at 6 rates of 0, 0.5, 1, 1.5, 2 and 4% (elemental P basis had the following chemical properties: P_2_O_5_-40.3%; CaO-54.6%, MgO-0.26%; cadmium-2.2 mg kg^−1^, chromium-18.05 mg kg^−1^, copper-5.85 mg kg^−1^, lead-6.05 mg kg^−1^ and zinc-13.22 mg kg^−1^) as ground RP with the aim of improving the P contents of the mixtures. Each was thoroughly mixed with enough feedstock (5 kg on dry weight basis) and prepared by mixing 2.16 kg of shredded paper and 2.84 kg of cow dung to achieve an optimized C:N ratio of 30. The resultant mixtures were loaded into worm boxes whose capacity measured 0.50 m × 0.40 m × 0.30 m (length × width × depth) with an exposed top surface area of 0.2 m^2^ in a well-ventilated farm building at an average mean ambient temperature of about 25 °C. Mature *E. fetida* earthworms were introduced into the vermin-reactors at a stocking density of 12.5 g worms kg^−1^ feed established by (Unuofin and Mnkeni 2014) as optimum for the bio-conversion of cow-dung paper-waste mixtures. Treatments were arranged in a completely randomized experimental design (CRD) with three replications. Moisture content in each box was brought to 80% by sprinkling water (Reinecke and Venter 1985) and maintained at this level by regular sprinkling throughout the 6 weeks of vermicomposting period.

Sample collection was carried out on days 0, 14, 28, 42 and 56 of vermicomposting and analysed for volatile solids (where ash content is a complementary parameter of VS) total C, total N, organic and inorganic phosphorus.

#### Physico-chemical analyses

The resultant vermicompost samples were analysed for volatile solids (VS), ash content, total carbon and total nitrogen, pH, extractable phosphorus P and humic substances. These vermicompost samples were first air-dried until constant weight was achieved and subsequently pulverized (<2 mm) to offer a uniform sample for analysis. The volatile solids (VS) were determined as sample weight loss of previously oven-dried (at 105 °C) samples following ashing at 550 °C for 4 h in a muffled furnace (Ndegwa et al. 2000) while total nitrogen (N) and carbon (C) were determined using a Truspec CN Carbon/Nitrogen analyser (LECO 2003). The pH was determined in a vermicompost-water suspension (1:2.5) with a pH/Conductivity meter as described by (Ndegwa et al. 2000).The suspensions were allowed to stand for 1 h after constant shaking using a mechanical shaker at 230 rpm for 30 min prior to pH measurement.

Total phosphorus was determined by digesting 0.5 g of air-dried composts samples in a MARS 5 microwave digester (CEM Corporation, Matthews, North Carolina) using aqua regia followed by the determination of phosphorus concentration in the digests by means of the reduced phosphormolybdenum blue method on a continuous flow analyser (San 2++ Skalar Continuous Flow Analyser, Skalar Analytical B.V. the Netherlands). Humic substances were determined following extraction, as described by Del Carmen et al. (2006). To the composted samples, 0.1 M NaOH (1:20 w/v ratio) was added and continuously shaken on a horizontal shaker for 4 h, followed by centrifugation of the resultant solution at 8000×*g* for 15 min. Afterward, the supernatants were separated into two portions: one portion was analysed for total extractable carbon fraction (C_EX_) through the Walkley and Black (1934) rapid titration method, as described by Anderson and Ingram (1996), and the remaining portion was adjusted to pH 2 using concentrated H_2_SO_4_ and set aside to coagulate for 24 h at 4 °C. The resulting precipitates that were formed comprising a humic acid-like carbon (C_HA_) while a fraction that remained in solution produced a fulvic acid-like carbon (C_FA_). The C_HA_ was calculated by subtracting the C_FA_ from the C_EX_. The humification indices were calculated using the following equations (Mupondi 2010):

1 $${\text{Humification ratio }}({\text{HR}}) = \left( {\frac{CEX}{C}} \right) \times 100$$

2 $${\text{Polymerization index }}\left( {\text{PI}} \right) = \left( {C_{\text{HA}} /C_{\text{FA}} } \right) \times 100$$

3 $${\text{Humification index }}({\text{HI}}) = \left( {\frac{CHA}{C}} \right) \times 100$$

#### Microbial biomass carbon

The initial microbial biomass carbon (MBC) content and that of the resulting vermicompost at day 56 were determined by means of the chloroform fumigation and extraction method (Vance et al. 1987). The process involved using 2 g of (oven-dry basis) moist compost which was, afterwards, fumigated for 24 h at 25 °C with ethanol free chloroform. Following the removal of fumigant, the sample was then extracted with 60 ml of 0.5 M potassium sulphate solution. The measurement of the organic C content in the extracts was done through dichromate oxidation Kalembasa and Jenkinson (1973), and the microbial biomass carbon (MBC) was calculated by means of the equation:

4 $${\text{MBC}} = {\text{E}}_{\text{C}} /{\text{K}}_{\text{EC}}$$

where E_C_ represents the organic carbon extracted from fumigated soil minus organic carbon that was extracted from (un)fumigated soil; K_EC_ (the proportion of the microbial C that is extracted from the compost) = 0.38 (Vance et al. 1987).

#### Morphological assessment of the resultant vermicomposts

Scanning electron microscopy (SEM) images of the samples were taken using a scanning electron microscope model JOEL (JSM-6390LV, Japan). Briefly, the samples were oven-dried and ground to pass through a 2 mm sieve. A small representative portion of the samples was coated with gold and mounted on SEM. Samples were then imaged by scanning them with a high-energy beam of electrons in a raster scan pattern.

#### Phytotoxicity study

Phytoxicity was determined, as described by (Unuofin and Mnkeni 2014). Briefly, two pieces of Whatman^®^ filter paper were placed inside a sterilized petri dish and dampened with the vermicompost extracts. Ten seeds of each crop species were placed on top of the filter paper and incubated for 5 days in the dark. A control was included for each crop species in which the filter papers were dampened with distilled water. Seed germination index (GI), relative seed germination (RSG) and relative root elongation (RRE) were calculated as follows:

5 $${\text{RSG}}\left( \% \right) = \frac{{{\text{Number}}\;{\text{of}}\;{\text{seeds}}\;{\text{germinated}}\;{\text{in}}\;{\text{the}}\;{\text{sample}}\;{\text{extract}}}}{{{\text{Number}}\;{\text{of}}\;{\text{seeds}}\;{\text{germinated}}\;{\text{in}}\;{\text{the}}\;{\text{control}}}} \times 100$$

6 $${\text{RRE}}\left( \% \right) = \frac{{{\text{Mean}}\;{\text{root}}\;{\text{elongation}}\;{\text{in}}\;{\text{the}}\;{\text{sample}}\;{\text{extract}}}}{{{\text{Mean}}\;{\text{root}}\;{\text{elongation}}\;{\text{in}}\;{\text{the}}\;{\text{control}}}} \times 100$$

7 $${\text{GI}}\left( \% \right) = \frac{{\left( {\% {\text{ Seed germination}}} \right) \times \left( {\% {\text{ Root elongation}}} \right)}}{100}$$

### Statistical analysis

Data reported herein are the means of three replicates (n = 3). Statistical analysis was done using the repeated measures analysis of variance (ANOVAR) since sampling was done non-destructively. Fisher’s protected least significant difference (*LSD*) test at P < 0.05 was used for means separation. All statistical analyses were done using JMP^®^ Release 10.0 statistical package (SAS Institute, Inc., Cary, North Carolina, USA, 2010)

## Results

### Effects of RP rate on volatile solids contents

Vermicomposting resulted in increases in nitrogen concentrations and decreases in volatile solids, total C and C:N ratio (Table 1). However, substantial amounts of organic matter (VS > 72%) remained in the final composts (Table 2). Carbon loss during composting was substantial. From 45 to 74% of the total C was lost from the cow-dung waste paper mixture (Table 1).

**Table 1:** Repeated measures ANOVAR for C:N ratio, HI, Total P, Total N, Bray-1 extractable P, MBC, VS and Ash contents, NH_4_-N and NO_3_-N

	Effect					
	Added RP rates		Time		Added RP rates × time	
	F_(5, 60_)	P	F_(4, 60)_	P	F_(20, 60)_	P
C:N ratio	1023.3	<0.0001	22,341.2	<0.0001	460.3	<0.0001
HI (%)	425.1	<0.0001	2357.6	<0.0001	101.7	<0.0001
Total P (g Kg^−1^)	2332.1	<0.0001	1064.1	<0.0001	894.4	<0.0001
Total N (%)	201	<0.0001	540	<0.0001	25	<0.0001
Bray 1P (mg Kg^−1^)	1895.5	<0.0001	1637.5	<0.0001	1034	<0.0001
MBC (mg Kg^−1^)	100.5	<0.0001	10,224.7	<0.0001	58.7	<0.0001
VS (%)	314.2	<0.0001	903.4	<0.0001	17.9	<0.0001
NH_4_^+^-N (mg Kg^−1^)	20.5	<0.0001	701.7	<0.0001	8.2	<0.0001
NO_3_^−^-N (mg Kg^−1^)	6.1	<0.0001	1003.7	<0.0001	23.3	<0.0001
NH_4_^+^:NO_3_^−^	3.36	<0.0001	230.2	<0.0001	5.6	<0.0001

*C:N* carbon to Nitrogen ratio, *HI* Humification index, *VS* Volatile solid, *MBC* Microbial biomass carbon, *NH*_*4*_^+^-*N* Ammonium-nitrate, *NO*_*3*_^−^-*N* Nitrate-Nitrite, *NH*_*4*_^+^ *:NO*_*3*_^−^ Ammonium: nitrate ratio

F values and probabilities are shown for each effect. *P* < 0.05 in italics is significant. n = 3

**Table 2:** Effect of added RP application on %Ash conversion in relation to %VS

RP application rates (%)	Initial %Ash	Initial %VS	Final %Ash	Final %VS
0	23 ± 1.0	77 ± 1.0	32 ± 0.01	68 ± 0.01
0.5	23.33 ± 0.5	76.66 ± 0.5	40 ± 1.0	60 ± 1.02
1	22.66 ± 1.2	77.33 ± 1.2	42.33 ± 3.21	57.67 ± 3.24
1.5	23.33 ± 0.5	76.66 ± 0.6	48.66 ± 0.5	51.37 ± 0.6
2	23 ± 1.0	77 ± 1.01	44.66 ± 1.54	55.37 ± 1.54
4	23 ± 1.0	77 ± 1.01	44.66 ± 1.54	55.37 ± 1.55

All values are the mean and standard deviation of three replicates

The volatile solid decreased significantly with time at each added RP rate, with the 1% P rate of RP application resulting in consistently low VS content levels, however, the absolute control where no RP was added had the highest VS levels (Table 2). The other rates of RP application resulted in intermediate low VS contents which followed the order 4% P ≈ 2% P < 1.5% P > 0.5% P (Table 2). Final per cent VS content ranged from 50 to 60% in vermicompost where RP was added, while in the control, where no RP was added, the VS content was 68% (Table 2).

Changes in pH by RP application rates were low and not consistent for cow-dung paper-waste mixtures. (i.e. RP application rates may have probably influence the decrease in pH values to some extent when compared to the control (0% P) for cow-dung paper-waste mixtures. However, decline in pH in most cases after 2 weeks could be due to the degradation of the organic acid compounds. Lowering of the pH could also be as a result of NH_3_ volatilization and nitrification during composting process) Table 3. Vermicomposting decreased pH but its effect was more pronounced in the presence of RP at 1% RP application rate. Vermicomposting decreased pH of the mixtures from 8.87 ± 0.01 in control to 7.98 ± 0.01 in the absence of RP, but decreased to 7.33 ± 0.01, 7.64 ± 0.02, 7.68 ± 0.03, 7.77 ± 0.01 and 7.81 ± 0.01 in the presence of 1, 1.5, 2, 4 and 0.5% of RP, respectively.

**Table 3:** Effect of rate of RP application on the pH of cow-dung paper-waste vermicompost mixtures

RP application rates (%)	Initial pH of cow-dung paper-waste mixtures	Final pH of cow-dung paper-waste mixtures
0	8.87 ± 0.01	7.98 ± 0.01
0.5	8.85 ± 0.01	7.81 ± 0.02
1	8.82 ± 0.01	7.33 ± 0.01
1.5	8.83 ± 0.01	7.64 ± 0.02
2	8.84 ± 0.01	7.68 ± 0.03
4	8.85 ± 0.01	7.77 ± 0.01

All values are the mean and standard deviation of three replicates

#### Effect of RP rate on the carbon to nitrogen ratio

**Fig. 1:**
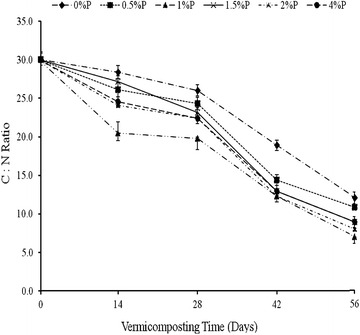
Effect of added P as rock phosphate (RP) rates and vermicomposting time on the C:N ratio of cow dung-waste paper mixtures during vermicomposting with *Esenia fatida*. *Bars* represent standard deviation

Both the added RP rates and time significantly affected the C:N ratio of cow-dung-waste paper vermicompost (Table 1). The C:N ratio decreased significantly with time at each added RP rate but pronounced differences between rates of RP application which were only observed up until day 28 beyond which, differences were minimal (Fig. 1). The greatest decrease in C:N ratio, on day 14, occurred where RP was added at a rate of 1%, from 30 to 18 while the least decline in C:N ratio (28) was observed where no RP was added.

Further decline in C:N ratio was observed beyond day 28 until termination of the experiment; however, the effects of added RP rates were not significantly different, except for 0 and 0.5% P on days 42 and 56, respectively. Final C:N declined ratios ranged from 6.9 to 10.6 in vermicompost where RP was added, while in the control, where no RP was added, the C:N ratio was 13 (Fig. 1). Consequently, the incorporation of RP accelerated the vermidegradation of cow-dung waste-paper mixtures during the early stages of vermicomposting up to 28 days, however, beyond this, the RP effect was minimal. Time required to vermicompost maturity was 56 days where no RP was added, nonetheless, it was 42 days or less in instances where RP was added (Table 4). The shortest time to vermicompost maturity was 33 days, and this was achieved through the application of 1% P as RP; this shortened the time to vermicompost maturity by 28% (Table 4). The C: N ratio of the final compost were: 1% P < 2% P < 4% P = 1.5% P < 0.5% P < 0% P (6 < 7 < 9 = 9 < 10 < 11 < 13) respectively. Indicating a decline from a starting C:N of 30:1 to a less than 10 in all RP added rates.

**Table 4:** Effect of rate of RP application on the vermidegradation time of cow-dung paper-waste mixtures as reflected by C:N ratio

Treat no.	Treatment % P as RP	Regression equations (C:N ratio VS Incubation time in days)	Days required to maturity (i.e. at C/N ratio = 12)	% Improvement in reducing time to vermicompost maturity
1	0	y = −0.0026x^2^ − 0.2072x + 30.8	46	
2	0.5	y = 0.002x^2^ − 0.1234x + 31.0	42	9*
3	1.0	y = 0.0066x^2^ − 0.7714x + 29.229	33	28*
4	1.5	y = 0.005x^2^ − 0.6458x + 31.6	41	11*
5	2.0	y = 0.0059x^2^ − 0.7038x + 30.4	41	11*
6	4.0	y = 0.007x^2^ − 0.7646x + 29	40	13*
		P > F	<0.0001	

* Relative to treatment 1 (where cow-dung paper-waste mixtures were only inoculated with *Eisenia fetida* and no RP addition)

#### Effect of RP rate on humification parameters

##### Humification index

**Fig. 2:**
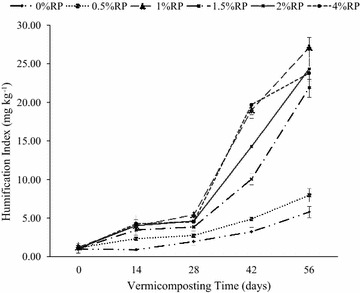
Effect of added RP rates and vermicomposting time on humification index. *Bars* represent standard deviation

The HI increased significantly with time (Table 1; Fig. 2) at each added RP rate (P < 0.0001) with no striking differences between rates of RP application up to 28 days (Fig. 2). However, wide differences on HI were observed on day 42 and 56 respectively (Fig. 2); this was consistent with the observed significant RP × time interaction (P < 0.0001) (Table 1). The differences on HI followed the order: 1% P = 4% P > 2% P > 1.5% P > 0.5% P > 0% P on day 42, whereas for day 56, the order was: 1% P > 2% P = 4% P = 1.5% P > 0.5% P > 0% (Fig. 2).

#### Effects of the rate of RP on MBC

**Fig. 3:**
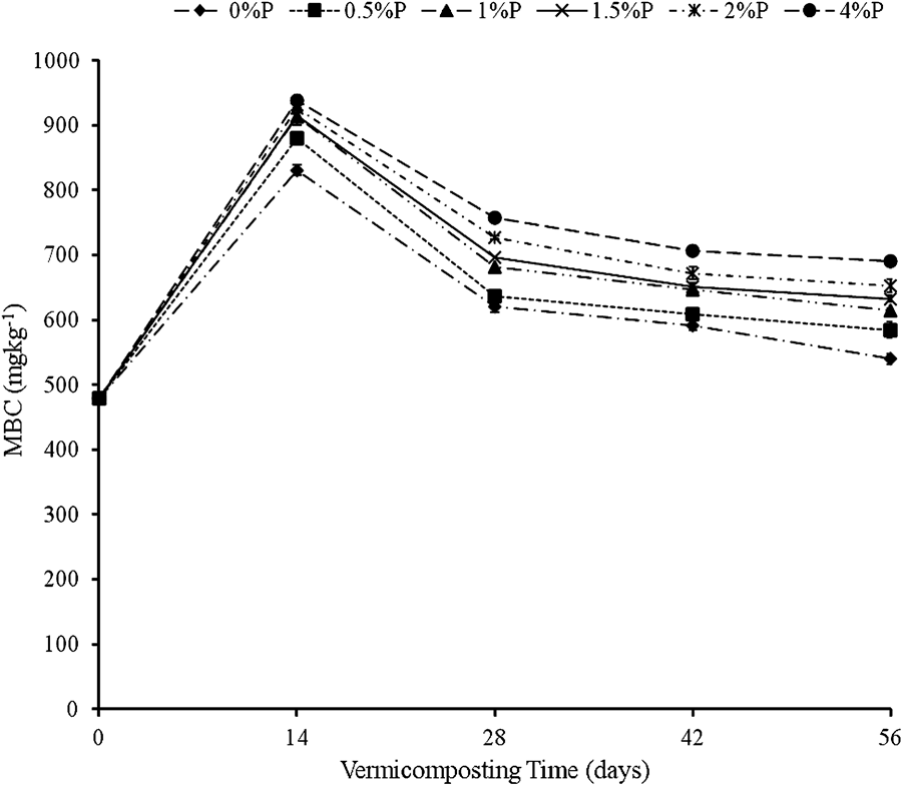
Effect of added RP rates and vermicomposting time on MBC. *Bars* represent standard deviation

Added RP had a significant effect on the MBC of cow-dung-waste paper vermicompost. Nevertheless, this effect varied with time as revealed by the significant interaction between RP rates and time (Table 1; Fig. 3). The MBC increased sharply up to day 14 at each added RP rate but declined sharply thereafter up to day 28 where-after the decline in MBC was gradual up to day 56 (Fig. 3). The level of MBC at each rate of RP application followed the order: 4% P > 2% P > 1.5% P ≈ 1.0% P > 0.5% P > 0% P (Fig. 3). The significant RP × Time interaction on MBC was largely due to the fact that initially, all RP treatments had the same level of MBC, but this changed as time progressed, thereby peaking at day 14 and declining thereafter.

#### Changes in the morphological structure of cow-dung-waste paper mixture during vermicomposting

**Fig. 4:**
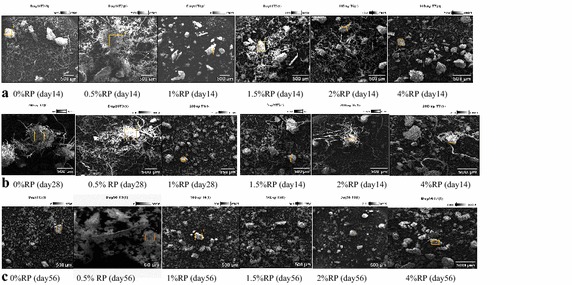
**a**–**c** Scanning electron microscope images showing the effect of added RP application rates and time on vermicompost morphological properties

Figures 4a–c show the effects of the rate of RP application on the morphological structure of cow-dung-waste paper mixtures during vermicomposting recorded using a scanning electron microscopy (SEM). The SEM images of the cow-dung-waste paper mixtures at the beginning of the experiment (not shown) showed compacted aggregates of cellulose and protein fibres. However, inoculation of earthworm and addition of RP facilitated the breakdown of the recalcitrant fibre in the paper and the cow dung to produce degraded vermicompost to varying degrees, depending on the rate of added RP and vermicomposting time (Fig. 4a–c). The SEM images show that degradation of the cow-dung-waste paper mixtures intensified with time at each rate of RP application. Wide differences in the level of degradation, at different rates of RP application, are seen on days 14 and 28. However, by day 56, the vermicomposting mixtures appeared to be equally degraded (visually) except where RP was added at a rate of 1% P where greater degradation was observed (Fig. 4a–c). On day 28, there were pronounced differences in the segregation of the wastes mixture aggregate particles which appeared to follow the order: 1% P > 4% P > 2% P > 1.5% P > 0% P > 0.5% P. Generally, the incorporation of RP at the rate of 1% P resulted in consistently greater vermidegradation of the cow-dung-waste mixtures on each sampling date than with the other RP treatments. This was reflected through higher segregation of the waste mixtures’ aggregate particles (Fig. 6 a–c).

#### Effects of RP rate on Nitrite-N NH_4_^+^-N and NH_4_^+^:NO_3_^−^ ratio

**Fig. 5:**
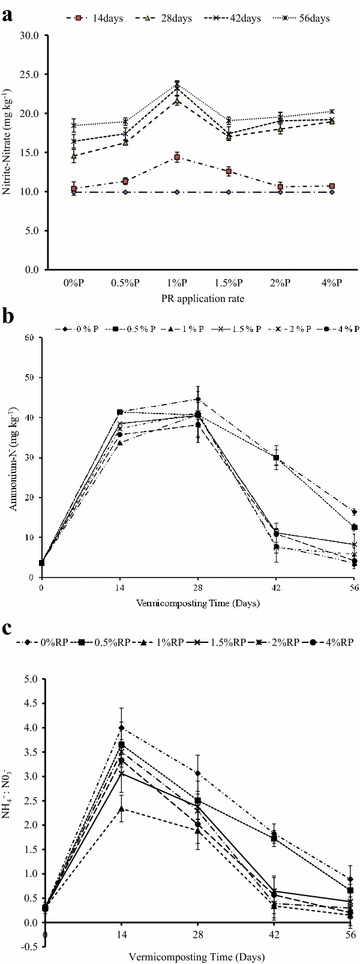
Effect of RP rates and vermicomposting time on **a** nitrate-N, **b** ammonium -N and **c** ammonium: Nitrate ratio

Nitrite-N, Ammonium-N and the NH_4_^+^:NO_3_^−^ ratio of cow-dung-waste paper vermicompost were significantly affected by added RP, however, these effects varied with time, as reflected by a significant interaction between RP rates and time (Table 1). The Nitrite-N content of the waste mixtures increased significantly with time at each added RP rate, with the 1% P rate of RP application resulting in consistently the highest Nitrite-N; the absolute control where no P was added had the lowest Nitrite-N (Fig. 5a). The other rates of RP application resulted in intermediate Nitrite-N contents which followed the order: 4% P ≈ 2% P ≈ 1.5% P > 0.5% P (Fig. 5a). The final Nitrite-N contents ranged from 35 to 19.1 mg kg^−1^ in vermicomposts where RP was added, while in the control, where no RP was added, the Nitrite-N was 18 mg kg^−1^ (Fig. 5a). The observed significant RP × Time interaction on Nitrite-N content was largely as a result of the fact that the Nitrite-N-content was the same for each RP treatment on day 0, but differences set in as time progressed. These differences between treatments were wide on days 28 and 42 but much narrower on day 56 except for the 1% P rate of RP incorporation which resulted in substantially higher values than the rest of the treatments (Fig. 5a).

The ammonium-N (NH_4_^+^-N) and the ratio of NH_4_^+^:NO_3_^−^ in cow-dung-waste paper vermicompost followed the same trend whereby they both increased up to day 14 with no striking differences in added RP, thereafter; they decreased linearly up to day 28. Beyond day 28, sharper and significant decreases were observed with time at each added RP rate (Fig. 5b, c). The NH_4_^+^-N and the NH_4_^+^:NO_3_^−^ ratios of the waste mixtures both increased significantly with time at each rate of added RP. The control, where no P was added, had the highest NH_4_^+^-N and the NH_4_^+^:NO_3_^−^ ratio, while 1% P rate of RP application resulted in consistently the lowest contents (Fig. 5b, c). The other rates of RP application followed the order: 4% P ≈ 2% P ≈ 1.5% P > 0.5% P (Fig. 5b, c). Final NH_4_^+^-N contents and the NH_4_^+^:NO_3_^−^ ratio values ranged from 12 to 4 mg kg^−1^ and 0.8 to 0.1 where RP was added, respectively (Fig. 5b, c). In the control where no RP was added, the NH_4_^+^-N content and the NH_4_^+^: NO_3_ ratio were 16.4 mg kg^−1^ and 0.9, respectively (Fig. 5b, c).

#### Effect of rate of RP on the phytotoxicity level

Three parameters [relative root elongation (RRE), relative seed germination (RSG), and germination index (GI)] were used to evaluate the phytotoxicity of the final vermicomposts using tomato, carrot and radish as test crops. All three parameters were significantly (P < 0.05) influenced by the added RP (Table 5). For each test crop, the three indices for phyto-toxity increased with the rate of RP application, peaking at the 1% P rate of RP application and declining, thereafter, with each increment in RP application (Table 5). The GI ranged from 90 to 138, 93 to 151 and 92 to 152 for tomato, carrot and radish, respectively. The highest GI was realized at the 1% P rate of RP application, which was significantly different from the other RP treatments (Table 5).

**Table 5:** Effect of added RP on the phytotoxicity of cow-dung waste-paper vermicomposts

Treatment	Tomato			Carrot			Radish		
	RRE (%)	RSG (%)	GI (%)	RRE (%)	RSG (%)	GI (%)	RRE (%)	RSG (%)	GI (%)
0% P	110d	84d	90e	96c	93e	93f	86d	108e	92e
0.5% P	116c	92c	108d	102b	100d	102e	91c	117d	107d
1% P	124a	110a	138a	109a	135a	151a	101a	150a	152a
1.5% P	120b	100b	120c	107a	110c	116d	95b	125c	119c
2% P	121b	102b	125bc	104a	115c	127c	99a	125c	122c
4% P	126a	102b	129b	108a	125b	140b	99a	134b	132b
CV (%)	5	9	10	13	7	5	4	2	4
*P* value	<. 028	<. 028	<. 028	<. 028	<. 028	<. 028	<. 028	<. 028	<. 028

Numbers followed by different letters in each column are significantly different according to the LSD test at P < 0.05

*RRE* Relative root elongation, *RSG* Relative seed germination, *GI* Germination index

#### The effect of RP rate on total P, Bray 1 extractable P

**Fig. 6:**
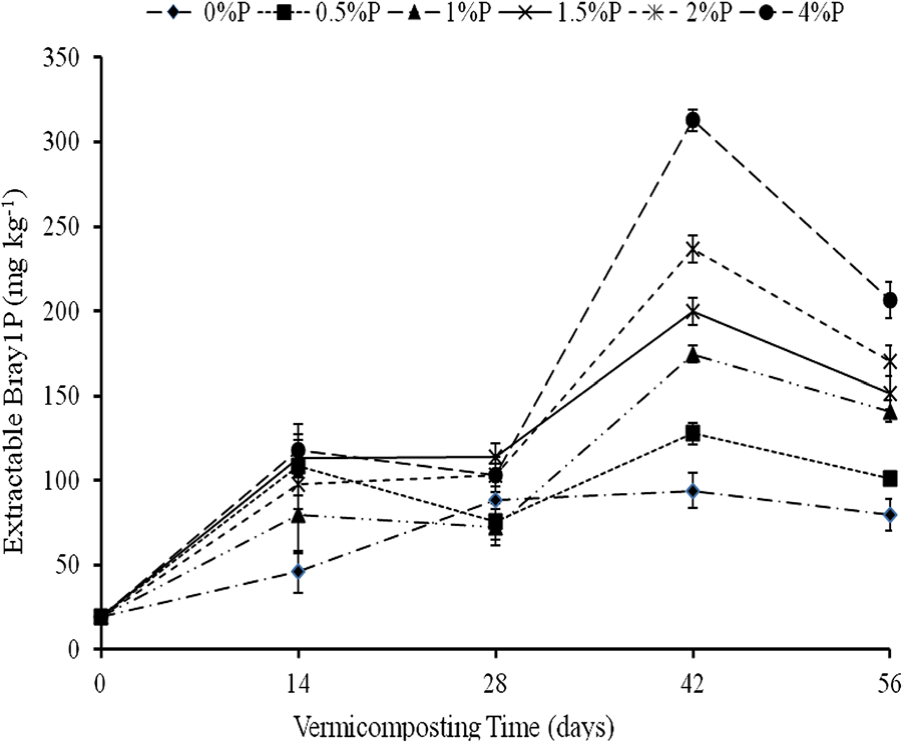
Effect of added RP rates and vermicomposting time on Bray 1 extractable P during vermicomposting of cow-dung-waste paper mixtures. *Bars* represent standard deviation

Addition of RP to cow-dung-waste paper mixtures resulted in increase in both total P and Bray 1 extractable P contents with each increment of added RP (Table 6). The total P content of the final vermicomposts ranged from 0.18% where no RP was added to 2.31% P when RP was added at a rate of 4% P. Corresponding values for Bray 1 extractable P were 80 to 207 mg P kg^−1^ (Table 6). The Bray-1 P content of the waste mixtures increased significantly with time at each rate of added RP rate; consequently, the 4% P rate of RP application was consistently in the highest Bray-1 P, and the absolute control where no P was added had the lowest Bray-1P (Fig. 6). This increased sharply up to day 14 at each added RP rate but declined sharply thereafter up to day 28. Beyond day 28, there was a sharp increase in Bray-1 P up to day 42 followed by a gradual decline up to day 56 (Fig. 6).

**Table 6:** Effects of added RP on Total P and Bray-1 extractable P contents of the final cow-dung paper-waste vermicomposts

Added RP	Total P (%)	Bray 1 P (mg kg^−1^)	Increase in Extractability of P (%)
0% P	0.18	80	
0.5% P	0.55	101	26
1% P	0.97	141	76
1.5% P	1.32	152	90
2% P	1.76	170	113
4% P	2.31	207	159
CV	5	3	
P value	<.0001	<.0001

## Discussion

### Effects of RP rate on selected maturity

One of the main aims of this study was to explore the possibility of enhancing the bio-transformation of cow-dung-waste paper mixtures with lower rates of RP integration.

The observed significant decrease in volatile solids contents with time, at each rate of added RP (Table 2), indicated degradation of organic matter (OM) in the vermicomposting mixtures, as reported by Mupondi (2010). This trend was confirmed by the maturation parameters used to monitor the stabilization of the vermicomposts, namely: Humification index (HI) (Fig. 2), microbial biomass carbon (MBC) (Fig. 3), Nitrite-N (Fig. 5a), Ammonium-N (Fig. 5b), and ratio of NH_4_^+^:NO_3_^−^ (Fig. 5c) and SEM imagery (Fig. 4a, c).

Vermicomposting decreased pH of the mixtures from 8.87 ± 0.01 in control to 7.33 ± 0.01, of different RP application rates, respectively. This is in accordance with the results of (Atiyeh et al. 2000; Venkatesh and Eevera 2008; Raphael and Velmourougane 2011). pH decrease may be due to the nitrification process experienced by the composting process, accumulation and reduction of organic acids from microbial metabolism, production of fulvic and humic acids during decomposition or as a result of NH3 volatilization during composting process (Albanell et al. 1988; Hanc and Vasak 2015; Cáceres et al. 2006).

The C:N ratio results (Fig. 1) confirmed that incorporation of RP accelerated the vermidegradation of the cow-dung-waste paper mixtures in that by day 42, the C:N ratio of the waste mixtures had declined from 30 when the experiment was initiated (day 0) to less than 14 where RP was incorporated, compared with 18 (where it was not). The results further revealed that the improved degradation was fastest when RP was added at a rate of 1% P, which allowed compost maturity to be achieved within 33 days. This indicated that a rate lower than the lowest rate of 2% P as RP used by Mupondi (2010) could be used to improve the rate of biodegradation of cow-dung-waste paper mixtures using *E. fetida*. The possibility of using lower rates of RP incorporation to enhance the vermidegradation of cow-dung-waste paper mixtures and (possibly) other waste will mean less transportation costs to places far from the Limpopo Province of South Africa where RP is mined.

The rate of C:N ratio decline was fastest between days 14 and 28 and slowest after day 42, thereby explaining the observed interaction between significant RP rate and time (Table 1). Bernal et al. (2009) and Lim et al. (2014) reported that the decline in C:N ratio could be attributed to the decomposition of organic matter as a result of microbial action. This is supported by the MBC data (Fig. 3) which shows that microbial activity was the highest on day 14 followed by day 28. This is a period coinciding with the sharpest decline in C:N ratio at each rate of added RP. This is further supported by the nitrate and ammonium data (Fig. 5a, b) which shows that ammonification and nitrification, both microbially-mediated processes, were most intense during this period. Ammonification is produced before nitrification. In this process, ammonia is converted into nitrite and nitrate during the nitrification process, for which mainly two different groups of microorganisms are responsible, the ammonia oxidizing and the nitrite-oxidizing bacteria. The nitrate is further assimilated into organic material or reduced to nitrogen oxides by denitrifying bacteria or completely into ammonia by the process of nitrate ammonification, mainly operated by fermentative microorganisms. The MBC increased with the rate of RP application, thus suggesting that RP had a stimulatory effect on microbial activity. Mupondi (2010) reported a similar trend of highest microbial activity on day 14 followed by day 28 during the vermicomposting of dairy manure-paper waste mixtures, as well as enhanced increase in available P. The author ascribed this increase in available P to the presence of high microbial populations, as shown by large amounts of microbial biomass C and P at the early vermicomposting stage. This increase, the author further noted, could be as a result of an increase in phosphate-solubilising microorganisms. Also the gut of the earthworm usually produces a considerable amount of alkaline phosphatase which is essential enzyme excreted through cast deposition and involved in biogeochemical cycle of phosphorus in soils (Lim et al. 2011). Similarly, Bhattacharya and Chattopadhyay (2002) reported an increase in phosphate-solubilising microorganisms while vermicomposting fly-ash amended cattle manure.

The increase in the HI with time indicated that the vermidegradation increased humification of the cow-dung-waste paper mixtures. This transformation was influenced by the rate of RP application and interestingly, it was highest at the 1% P rate of RP application. This intensification of degradation of the mixtures was further confirmed by the images from the scanning electron microscopy (Fig. 4a, c) that showed RP application intensified the degradation of cow-dung-waste paper mixtures and that the 1% P rate of RP application resulted in consistently greater vermidegradation of the waste mixtures at each sampling date than with the other RP treatments. This was further reflected in higher segregation of the waste mixture aggregate particles. According to Lim and Wu (2015, 2016) the SEM image of the vermicompost showed a distinct physical appearance that was more scattered fragmented and smaller in nature as compared to the initial waste mixtures. Therefore, the SEM images observed in this study were similar to Lim and Wu (2015, 2016) and showed that degradation of the cow dung-waste-paper mixtures was intensified with time at each rate of RP application. The wide differences in the extent of degradation, at different rates of RP application observed on days 14 and 28 (pH 20, which coincided with the period of maximum microbial activity (Fig. 3), is further proof that the observed improved vermidegradation of waste mixtures mixed with RP could be related to its stimulatory effect on soil microorganisms. The RP constituent responsible for this stimulation remains to be established.

The seed germination index is a more direct indicator of both vermicompost and compost maturity as it directly tests whether the finished vermicompost can inhibit plant growth or not when used as a growth media. The over 80% GI observed for all test crops in this study indicated that addition of P as RP to cow-dung-waste paper mixtures in the presence of *E. fetida* resulted in vermicompost that was free of phytotoxins, according to (Zucconi et al. 1981; Tam et al. 1998). These results are also in agreement with those of Bustamante et al. (2001) which showed that a germination index of ≥80% indicated the disappearance of phytotoxins in composts.

#### Effects of RP rate on the P enrichment

The total P content of 2.31% P in the final cow-dung-waste paper vermicompost, where RP was added at a rate of 4% P, is consistent with results of Biswas and Narayanasamy (2006) who reported increase in the total P content of straw compost from 0.37% in control to 2.20% by applying 4% P as RP. Yan et al. (2012) also reported an increase in the total P of rice straw compost from 0.392 to 0.82% through application of 2% P as RP. More significantly, however, in the present study, Bray 1 extractable P increased from a low 80 mg P kg^−1^ where no RP was added to 207 mg P kg^−1^ where RP was added at a rate of 4% P (Table 5). This contributed to the increase in the extractable P and, thus, implies significant increases in the effectiveness of vermicompost in providing available P.

The improvement in the extractability of P with the rate of RP application (Table 5) mirrored an increase in microbial biomass with the rate of RP application (Fig. 3), thereby suggesting that among the micro-organisms stimulated by added RP were phosphate bacteria which facilitated the mineralization, hence, extractability of P from the added RP. The P release is mediated by phosphate enzymes produced by micro-organisms in the earthworm guts and those in the earthworm casts (Yan et al. 2012). The observed increase in humic acids reflected by the humification index or polymerization index (HI or PI) (Fig. 2) could also account for the enhanced mineralization and dissolution of P. According to Singh and Amberger (1990), humic acids can absorb significant quantities of calcium ions and release an H+ ion, which further facilitates the dissolution of RP. In addition, the functional groups in humic acids such as carboxylic and phenolic groups can also chelate Ca^++^ ions, thus providing a driving force for the mineralization and dissolution of P from RP.

The increase in Bray 1 P up to day 42 and its decline thereafter (Fig. 6) suggested that after 42 days, the mineralized P underwent precipitation reactions, hence the decreased extractability. Sequential extraction showed that the H_2_O-Pi fraction made the largest contribution to the total inorganic P extracted, thus suggesting that most of the mineralized P, during the vermicomposting of the cow-dung-waste paper mixtures enriched with RP, would be available for plant uptake. These results indicate that vermicomposting cow-dung waste-paper mixtures enriched with phosphate rock improves the solubilisation of the Phalaborwa RP used in this study and thus improved its fertilizer value. According to Edwards et al. (2010), RP is an acceptable source of P for organic agriculture, but its use is limited by its slow rate of P release. The high total P and extractable P contents observed in the cow-dung waste-paper vermicomposts enriched with RP point to their potential as organic P fertilizers. Future studies should explore this potential.

## Conclusions

The results of this study have confirmed that incorporation of rock phosphate improves the biodegradation of cow-dung-paper-waste mixtures. The cause of the influence of P is as a result of the gut of the earthworm which produces a considerable amount of alkaline phosphatase, which is an essential enzyme excreted through cast deposition and involved in biogeochemical cycle of phosphorus in soils. The result further revealed that optimal vermidegradation can be achieved with the application of 1% P as RP. It also showed that the final C/N ratio at 14, 28 and 33 days of vermicomposting were 20, 17 and 12 respectively. Hence, at this rate of RP incorporation, the vermicomposting mixtures required only 33 days to reach maturity. The improvement in vermicomposting occurred mostly between days 14 and 28. Although a 1% P rate of RP application is all that was needed for fast maturation of the vermicompost, higher rates of RP application are necessary for an enhanced P fertilizer value of the resultant vermicompost. Therefore, higher rates of RP incorporation may be necessary where final composts with higher P contents and, thus, better P fertilizer value are desired. Future studies will need to examine the agronomic value of these composts, find out the reason why 1% treatment was the one with the best degradation of the OM. Nevertheless, the results of the present study have shown that cow-dung waste-paper vermicomposts enriched with phosphate rock have potential as organic fertilizers which would be acceptable in organic farming.
